# Computer vision detects inflammatory arthritis in standardized smartphone photographs in an Indian patient cohort

**DOI:** 10.3389/fmed.2023.1280462

**Published:** 2023-11-09

**Authors:** Sanat Phatak, Somashree Chakraborty, Pranay Goel

**Affiliations:** ^1^KEM Hospital Research Centre, Pune, India; ^2^Indian Institute of Science, Education and Research, Pune, India

**Keywords:** artificial intelligence, inflammatory arthritis, digital health, computer vision, screening

## Abstract

**Introduction:**

Computer vision extracts meaning from pixelated images and holds promise in automating various clinical tasks. Convolutional neural networks (CNNs), a deep learning network used therein, have shown promise in analyzing X-ray images and joint photographs. We studied the performance of a CNN on standardized smartphone photographs in detecting inflammation in three hand joints and compared it to a rheumatologist’s diagnosis.

**Methods:**

We enrolled 100 consecutive patients with inflammatory arthritis with an onset period of less than 2 years, excluding those with deformities. Each patient was examined by a rheumatologist, and the presence of synovitis in each joint was recorded. Hand photographs were taken in a standardized manner, anonymized, and cropped to include joints of interest. A ResNet-101 backbone modified for two class outputs (inflamed or not) was used for training. We also tested a hue-augmented dataset. We reported accuracy, sensitivity, and specificity for three joints: wrist, index finger proximal interphalangeal (IFPIP), and middle finger proximal interphalangeal (MFPIP), taking the rheumatologist’s opinion as the gold standard.

**Results:**

The cohort consisted of 100 individuals, of which 22 of them were men, with a mean age of 49.7 (SD 12.9) years. The majority of the cohort (*n* = 68, 68%) had rheumatoid arthritis. The wrist (125/200, 62.5%), MFPIP (94/200, 47%), and IFPIP (83/200, 41.5%) were the three most commonly inflamed joints. The CNN achieved the highest accuracy, sensitivity, and specificity in detecting synovitis in the MFPIP (83, 77, and 88%, respectively), followed by the IFPIP (74, 74, and 75%, respectively) and the wrist (62, 90, and 21%, respectively).

**Discussion:**

We have demonstrated that computer vision was able to detect inflammation in three joints of the hand with reasonable accuracy on standardized photographs despite a small dataset. Feature engineering was not required, and the CNN worked despite a diversity in clinical diagnosis. Larger datasets are likely to improve accuracy and help explain the basis of classification. These data suggest a potential use of computer vision in screening and follow-up of inflammatory arthritis.

## Introduction

Artificial intelligence is rapidly changing the landscape of healthcare in general and offers promising applications in automating screening and follow-up of chronic diseases (1). Deep-learning methods encapsulate the use of artificially created neural networks to learn functions that describe them (2). Specifically, computer vision focuses on the extraction of meaning from pixelated images and videos. A convolutional neural network (CNN) is a type of deep-learning neural network designed specifically for computer vision tasks. Using the mathematical operation called convolution enables a CNN to extract image features that are essential for image recognition, classification, and segmentation (3). Transfer learning is a technique that adapts a network which has previously been trained on a large dataset to effectively train that network on the images at hand.

CNNs are relatively recent but already offer the potential to automate various human tasks in healthcare. Most commonly, CNNs have been used to classify radiological images into different patterns (image classification) that allow faster and more accurate diagnoses (4). A CNN (CheXNet) successfully detected cases of pneumonia on chest radiographs (5). In addition, CNNs are useful in segmenting medical images, helping doctors identify important features such as malignant tumors and inflammatory lesions. A CNN to help diagnose diabetic retinopathy recently received Food and Drug administration (FDA) approval (6). As computer vision techniques become more refined, each field of medicine and healthcare is likely to benefit.

The application of computer vision in inflammatory arthritis is nascent and has mainly focused on imaging results, such as predicting erosive arthritis on X-rays, thus taking strides in automating scoring used in clinical trials (7). It has successfully been applied to augment imaging-based diagnosis in rheumatology, including the detection of spondyloarthritis on sacroiliac joint MRI (8). It has also been utilized to automate disease activity scoring on joint ultrasound in RA (9). They are particularly useful in measuring structures, such as the median nerve area in carpal tunnel syndrome (10) and cartilage thickness in joints (11).

Inflammatory arthritis presents with joint pain and swelling and is typically diagnosed by a physician through medical history and examination during a visit. Detecting and recording the number of swollen joints is included in most classification criteria and disease outcome measures in these diseases (12, 13). Apart from deeply seated joints such as the hip, most joints with synovitis do have visible signs of inflammation, including redness and swelling. We believed that these features would be amenable to detection by computer vision on standardized mobile phone photographs of joint areas. A recently published CNN could distinguish inflammation in the proximal interphalangeal (PIP) by recognizing the obliteration of creases on the joint (14). Our ongoing study evaluates standardization methods for photography as well as the accuracy of computer vision in identifying inflamed joints. Subject to further validation, such approaches could be valuable digital additions for screening programs in communities with limited access to rheumatology specialists, in addition to longitudinal follow-up studies and clinical trials (15).

We present preliminary data on the performance of a CNN in detecting synovitis in a few selected joints for our ongoing study. We expanded our analysis to three joints, the wrist, the second/index finger proximal interphalangeal (IFPIP), and the third/middle finger PIP (MFPIP), and selected them based on a relatively higher prevalence of involvement in the dataset. We evaluated the performance of this CNN without incorporating feature engineering.

## Methods

### Patient selection

We enrolled patients from the rheumatology department’s outpatient clinic located at KEM Hospital in Pune as well as from a private rheumatology practice in Pune, India. We included consecutive patients with inflammatory arthritis involving the hand joints for less than 2 years since symptom onset. Cases of long-standing arthritis were excluded from this dataset to prevent confounding with joint deformities. Permissible clinical diagnoses included but were not limited to rheumatoid arthritis (RA), psoriatic arthritis (PsA), systemic lupus erythematosus (SLE), Sjogren syndrome (SS), and chronic viral arthritis. RA was classified according to the American College of Rheumatology/European Alliance of Associations of Rheumatology (ACR/EULAR) 2011 criteria (12), PsA according to the Classification for Psoriatic Arthritis (CASPAR) criteria (16), and SLE according to the Systemic Lupus International Collaborating Clinics (SLICC) classification criteria (17); all others were clinical diagnoses made by the rheumatologist. We excluded patients with visually appreciable deformities, co-existing nodal osteoarthritis, and systemic sclerosis.

Demographic details recorded included age and gender; clinical details included duration and diagnosis of the disease as well as extra-articular features, especially the involvement of coexistent skin lesions on the hands. The patient’s and doctor’s global perception at that clinical visit (on a visual analog scale) and results of markers of inflammation (ESR, CRP, or both) were recorded. An independent clinical examination was conducted by a trained rheumatologist. The presence of synovitis was recorded as a binary (yes/no) opinion, based on inspection, palpation, and range of motion testing in each of the 15 joints in both hands (four distal interphalangeal, four proximal interphalangeal, one interphalangeal of thumb, five metacarpophalangeal, and one wrist).

### Hand photograph acquisition and standardization

Photographs of both hands from the dorsal and palmar aspect were taken by a member of trained research staff in standardized conditions. We modified a foldable photo studio light box used for product photography of dimensions 26 × 26 × 26 cm with an inbuilt LED light system and white background to ensure uniform camera placement on the top. The modified studio box was kept on a desk, and patients sat in a chair to ensure appropriate placement of the hand. All photographs were taken using an iPhone 11 (Apple Inc., California, United States).

### Data entry and anonymization

A unique identity generator was used to create randomly generated identifiers (18). A total of two thousand unique IDs were generated for patients with arthritis. Each subject was assigned two IDs: a personal ID (IDP), linked to personal information of the patient, and a study ID (IDS), linked to photographs and clinical information. The key to linking these two is securely stored with SP. The photographs were labeled with the IDS and suffixed according to the hand’s laterality and view. Patient-identifying data were entered with the IDP and clinical data related to synovitis prevalence with IDS. Only the anonymized, labeled photographs and non-identifying information were shared.

### Joint detection and image cropping

**Figure 1:**
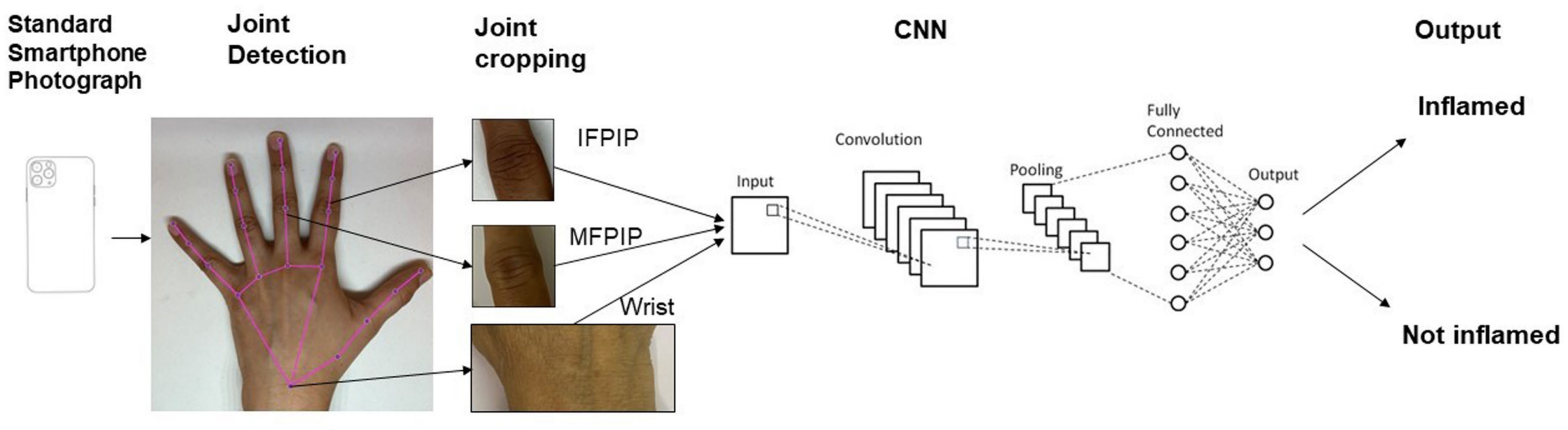
Schema of photograph processing and outputs from the convolutional neural network.

We used MediaPipe, an open-source library, for tracking the key points of the hands (Figure 1) (19). MediaPipe can locate the joints of interest in clinical images of the hands. The *x* and *y* coordinates of joints were extracted and used to isolate cropped images of the IFPIP, MFPIP, and wrists.

### Neural networks

The main model used for training was a ReNet-101 backbone modified for two class outputs: “Inflamed” or “not.” Transfer learning was used throughout. The dataset was split into a ratio of either 80:20 or 70:30 between training and validation sets. Typically, hyperparameters varied during each training run: batch sizes ranged from 32 to 64, training epochs were kept below 100, learning rate was on the order of 1e^−4^, solvers were either “adam” or “sgdm,” and early stopping was used when reasonable. Image augmentation typically included a random rotation of up to 20 degrees both clockwise and anticlockwise, a random scaling between 0.8 and 1.2, random reflection along the *x*-axis, and a random *x*- or *y*-shift of up to 10 pixels. Neural networks were developed using the Deep Learning Toolbox in MATLAB (MATLAB version: R2023b, Natick, Massachusetts: The MathWorks Inc.; 2023.)

### Hue adjustment for skin tone

**Figure 2:**
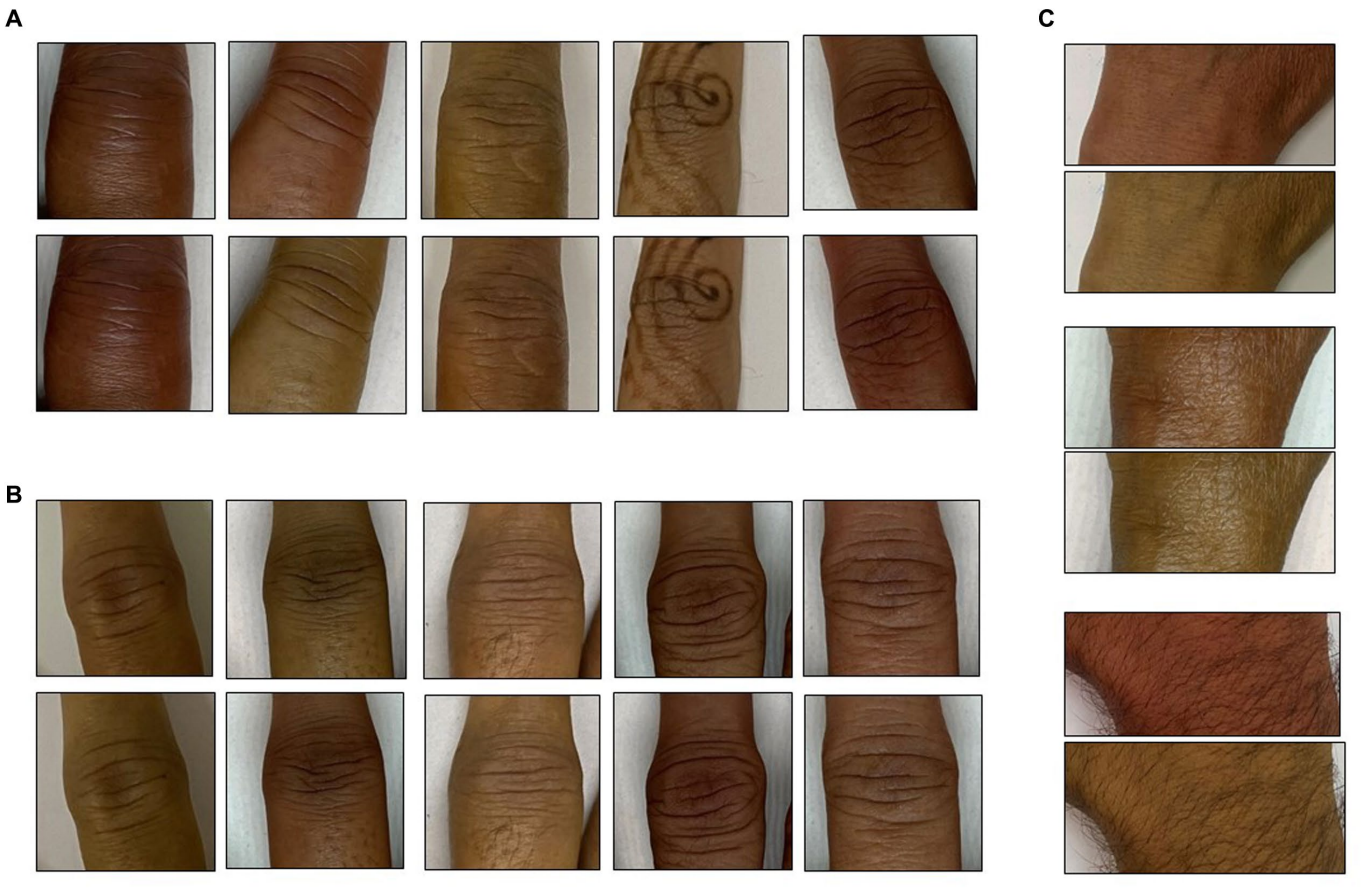
Hue augmentation for skin tone: representative hue-augmented pairs with random hue variation between 6 and 34 degrees. **(A)** Index finger proximal interphalangeal joints, **(B)** middle finger interphalangeal joints, and **(C)** wrist joints. Note the henna on some images did not change the results.

It is widely known that human skin varies in hue roughly between 6 and 34 degrees (Skin Colour Analysis. University of Edinburgh. 2001). A hue-augmented dataset was created as follows: the RGB image was converted to HSV format and a random hue component between 6 and 34 degrees was applied: (0.1–0.016) × (rand − 0.5). The modified image was clipped for hue values lying outside (0.16, 0.1). Each image was augmented for a hue change once (Figure 2).

### Statistics

Patient data, especially synovitis distribution, are presented as frequencies, mean (standard deviation) for normally distributed continuous variables, and median (IQR) for those that were not normally distributed. Normality was ascertained using the Shapiro–Wilk test. We report accuracy, sensitivity, and specificity of the CNN in detecting synovitis in the three individual joints within the validation set. The rheumatologist’s opinion was considered the gold standard for this analysis. Sensitivity (a/a + b, true positive fraction amongst all patients with disease) and specificity (c/c + d, true negative fraction in all patients without disease) were calculated using standard formulae. Accuracy was calculated as the overall probability of a correct classification (Sensitivity × Prevalence + Specificity × (1 − Prevalence)). We calculated positive and negative predictive values, using the prevalence rates of inflammation for each joint from the entire dataset (20).

### Ethics statement

This study received ethics permission from the KEM Hospital Research Centre Ethics Committee (KEMHRCEC/2018) and a waiver from the IISER Ethics Committee for Human Research (IEHCR/Admin/2021/006). A data sharing agreement has been signed between the institutions for this study. All patients signed an informed consent document with special permission for the storage of photographs in the photo repository for 30 years. This study has been registered with the Clinical Trials Registry of India (CTRI/2020/08/027129).

## Results

### Patient characteristics

We included 100 consecutive patients (22 men) with inflammatory arthritis with an onset period of less than 2 years. The mean age of the cohort was 49.7 (12.8) years. The selected patients had a mean weight of 65.1 (12.6) kg and a mean height of 160.1 (4.5) cm. The mean hemoglobin level was 11.4 (1.8) g/dL; all the above variables were normally distributed. The median Erythrocyte sedimentation rate (ESR) was 36 (IQR 32) mm/h and the median C-reactive protein was 10.3 (IQR 12.6) mg/dL. Sixty-eight patients were classified as RA, eight had peripheral spondylarthritis, six had PsA, and 13 had connective tissue diseases (three with SLE, four with SS, and six with undifferentiated or mixed connective tissue disease). Five patients had chronic post-viral arthritis.

Synovitis was present in only one hand of 19 patients, while bilateral involvement was observed in 81 patients. Of the 200 hands studied, 52 had one swollen joint, 33 had two swollen joints, 38 had three swollen joints, and 58 had polyarthritis (four or more). The wrist was the most common joint to be swollen (62.5% of hands) followed by the MFPIP (47%) and IFPIP (41.5%). DIP joint swellings were relatively rarer (Table 1). All patients were able to lay their hands flat inside the photo box, as deformities were excluded. Since these were all adult participants and the depth of the box was constant, the aspect ratio was not altered, and the images were distortion-free; thus, no editing was required.

**Table 1:** Patient characteristics: distribution of synovitis in the dataset (*n* = 100).

Swollen joints	Left hand (*n* = 100)	Right hand (*n* = 100)	Total (*n* = 200)
Wrist	61	64	125 (62.5%)
MCP 1	5	15	20 (10%)
MCP 2	14	23	37 (18.5%)
MCP 3	16	21	37 (18.5%)
MCP 4	5	3	8 (4%)
MCP 5	3	4	7 (3.5%)
IP 1	8	19	27 (13.5%)
PIP 2 (IFPIP)	41	42	83 (41.5%)
PIP 3 (MFPIP)	44	50	94 (47%)
PIP 4	17	21	39 (19.5%)
PIP 5	17	20	37 (18.5%)
DIP 2	1	2	3 (1.5%)
DIP 3	2	1	3 (1.5%)
DIP 4	2	0	2 (1%)
DIP 5	1	1	2 (1%)
No swollen joint	9	10	19 (9.5%)

IP, interphalangeal; PIP, proximal interphalangeal; DIP, distal interphalangeal; MCP, metacarpophalangeal.

### CNN performance

**Figure 3:**
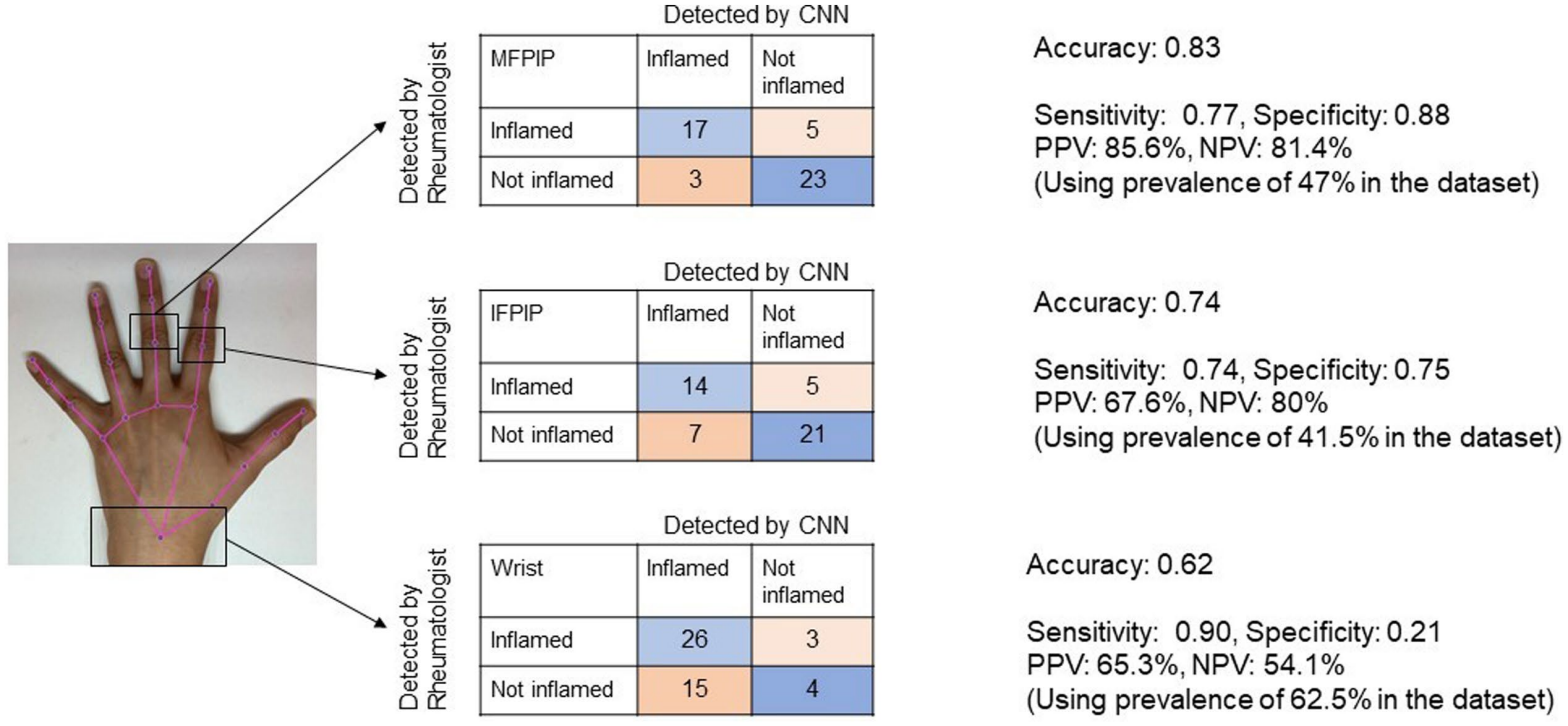
Representative image showing the performance of Convolutional Neural Network (CNN) for three joints in contingency tables. Accuracy denotes the overall probability that a patient is correctly classified. MFPIP, middle finger proximal interphalangeal joint; IFPIP, index finger proximal interphalangeal joint; PPV, positive predictive value; NPV, negative predictive value.

**Figure 4:**
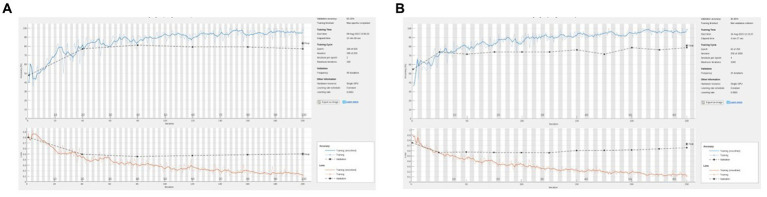
**(A)** Representative training graph for naive (no hue augmentation) middle finger PIP. On this run, accuracy = 83%, sensitivity = 77%, and specificity = 88%. **(B)** Representative training graph for naive (with hue augmentation) MFPIP. On this run, accuracy = 81%, sensitivity = 65%, and specificity = 92%.

The CNN achieved the highest accuracy with the MFPIP (83%), followed by the IFPIP (74%), and the wrist (62%) (Figures 3, 4). It should be noted that the percentages in the contingency table are largely representative. Given that the number of images in the dataset is relatively small for deep learning, particular training runs give rise to somewhat different accuracies, typically varying by approximately 5%, depending on the exact samples used in the training. Sensitivity was good in all three joints, but specificity was low at the wrist joint (Figure 3).

We created a dataset in which skin tone augmentation was applied in a 1:1 ratio, that is, each original image was augmented with one image with a modified hue (Figure 3). The training was carried out based on these data. The rationale was to augment the data with different samples of skin tones. We also attempted to train networks by employing a larger ratio of original to hue-modified images, which resulted in even higher validation accuracies. In principle, we were aiming for augmentation to increase the dataset; however, this may have simply introduced artifacts arising from having images (an original and its modified images) that are too similar between the training and validation sets. Finally, we chose to restrict to a conservative 1:1 ratio of original and hue-augmented images. The networks were trained with the training set containing hue-augmented images, while the validation set contained only the original images. With this training protocol, the results were comparable to the naive case above (without hue augmentation). In larger datasets, this is likely to be an interesting direction to pursue further.

## Discussion

In this proof-of-concept analysis, we demonstrated that computer vision was able to distinguish inflammatory arthritis, with reasonable accuracy in three selected synovial joints of the hand on standardized photographs. The CNN worked in a real-world setting despite the heterogeneity in the etiology of inflammation and without the need for feature engineering. The accuracy remained constant when using skin tone augmentation through modified hue. These data increase confidence in the utility of CNNs across different diseases and ethnic groups. We provide further credibility to the potential future use of applying computer vision on smartphone photographs as a screening and follow-up tool in inflammatory arthritis.

CNNs have been used previously in rheumatology, mainly in automating the analysis of microscopic immunofluorescence radiographic images, achieving the agreement close to human expert readers (21). A “cascade” of CNNs achieved accuracy comparable to trained rheumatologists in discriminating healthy joints from hand X-ray images (9). Similarly, CNNs have been trained to perform scores such as the modified van der Heijde Sharp Score for joint erosions on hand radiographs (22). A plethora of studies using both MRI and X-ray images in detecting and scoring rheumatoid arthritis erosions with various learning mechanisms and classification techniques are currently available (7). While these studies are highly useful, most of them are use cases for specialists once a diagnosis is made and require expensive imaging technologies. In more resource-limited settings such as ours, there is a need for applications at the screening and diagnosis levels. A preassessment using tele-health for efficient referral can help optimize time for rheumatologists (23). Smartphone-based photographs can be easily taken even before the patient has access to imaging technologies at negligible cost. Promising steps have been taken in establishing the utility of smartphone camera sensors in the assessment of physical function in RA. The PARADE study demonstrated their utility in capturing the functional ability in wrist movements and gait in patients (24). The innovative TELERA randomized trial evaluates the possibility of using app-based outcome measures in remotely following up with patients with arthritis (25).

Recently, a CNN recognized that joint swelling was shown to be efficacious as a digital biomarker (14). The authors used cropped-out images of the PIP joints in a similar-sized dataset as ours. The patient population in the study by Hugle et al. was more homogenous, with all patients satisfying the criteria for RA. However, there was no comment made regarding the joint-swelling distribution, ethnicity, and skin color characteristics of the cohort. It can be assumed that the population was predominantly Caucasian. All of our patients belonged to the South Asian ethnicity, and skin hues for these patients were categorized as grade 3–4 as per the Fitzpatrick classification. Our results expand on this literature by demonstrating that CNN accuracy is maintained not only on additional joints (IFPIP and wrist) but also across different diseases and ethnic groups, especially in darker skin tones where inflammatory erythema may not be as readily visible. Our photos included those with henna markings and hand jewelry, which did not reduce accuracy.

The CNN performance did not improve significantly by training on the hue-augmented images. Thus, we reason that, while hue augmentation is not an effective strategy for training, the results of the training (that is, the trained networks) will be effective for applying across populations with varying skin tones. Skin hue modification and its implications for training and prediction are likely to be an interesting area to pursue in future studies, especially to improve generalizability across populations.

Similar to any black box AI technology, we are not certain what parameters were used to classify synovitis by the CNN, and one can only speculate that color, shape, and contour changes were involved. Hugle et al. included the identification and extraction of dorsal finger folds from the joint images (10). Intuitively, this methodology may not extrapolate to synovial joints that do not have naturally occurring skin folds, such as the MCP joints, wrists, knees, or ankles. Our methodology ensures that any relevant features will be learned in the training. For example, it has learnt to ‘overcome’ confusion due to henna and rings. Even without feature engineering, our CNN achieved similar accuracy to their dataset, supporting a more general approach. The joint shape may be important since wrist inflammation was picked up less accurately in our analysis. Larger, uniform datasets would be required in order to delve deeper into attempting to explain the basis for the classification. All our images were standardized for device, background, and lighting. We surmise that standard acquisition and preprocessing/cropping steps reduce the variability in the image background that the CNN picks up. However, this may limit generalizability in the community using different devices. The final goal would be for patients to take photos themselves, and it would be very interesting to see how this affects the CNN performance.

Despite being an initial analysis of an ongoing collection, our study has certain strengths: our patient distribution is heterogeneous and reflects a real-world rheumatology practice. We used carefully standardized photographs using the same phone camera. One of the shortcomings of this study is that the dataset used for analysis is relatively small. This precluded an analysis of the other joints of the hand due to the low prevalence of synovitis. Our assessment of a rheumatologist’s assessment gold standard is also subjective. Further evaluation should include assessing the CNN’s performance against more sensitive measures, such as USG and MRI-detected synovitis. Finally, since we included only early arthritis, the performance in the presence of joint deformity or co-existing osteoarthritis and deformities, both important clinical confounders, cannot be commented upon at this stage. Distinguishing inflammation from deformity and including other commonly involved joints, such as the MCP, would be an important future endeavor. Nevertheless, our pilot results increase confidence that larger, good quality, and diverse datasets would improve the reliability of computer vision-based applications in inflammatory arthritis. These would also enable determining individual accuracies for different joints, and Bayesian methodologies could improve synovitis detection.

In conclusion, we have demonstrated that computer vision could detect synovial joint inflammation with reasonable accuracy, sensitivity, and specificity in standardized smartphone photographs of joint areas. With larger datasets, this technology has the potential to be a valuable remote tool in screening and follow-up of inflammatory arthritis.

## Data availability statement

The raw data supporting the conclusions of this article will be made available by the authors, without undue reservation.

## Ethics statement

The studies involving humans were approved by KEM Hospital Research Centre Ethics Committee. The studies were conducted in accordance with the local legislation and institutional requirements. The participants provided their written informed consent to participate in this study. Written informed consent was obtained from the individual(s) for the publication of any potentially identifiable images or data included in this article.

## Author contributions

SP: Conceptualization, Data curation, Formal analysis, Project administration, Resources, Writing – original draft, Writing – review & editing. SC: Data curation, Formal analysis, Writing – original draft. PG: Conceptualization, Formal analysis, Methodology, Software, Writing – original draft, Writing – review & editing.
